# Special Issue “Pediatric Diseases: From Molecular Mechanisms to Novel Therapeutic Strategies”

**DOI:** 10.3390/ijms27187976

**Published:** 2026-09-08

**Authors:** Elena Țarcă

**Affiliations:** Grigore T. Popa University of Medicine and Pharmacy, 700115 Iaşi, Romania; tarca.elena@umfiasi.ro

## 1. Introduction

Pediatric genetic diseases, congenital abnormalities, and childhood malignancies represent a diverse group of disorders that continue to challenge clinicians and researchers alike. Unlike adult diseases, pediatric disorders frequently arise from developmental, genetic, and epigenetic mechanisms that interact with dynamic physiological processes during growth [1,2]. Consequently, the diagnosis and management of these conditions require approaches specifically tailored to children rather than direct extrapolation from adult medicine. Recent progress in molecular biology, genomics, bioinformatics, and precision medicine has substantially improved our understanding of disease mechanisms, creating new opportunities for earlier diagnosis and individualized therapeutic interventions [1,3].

Despite these advances, significant knowledge gaps remain and although precision medicine has become a central objective of pediatric research, effective targeted therapies remain unavailable for many inherited or acquired disorders [4]. These limitations emphasize the continuing need for translational investigations that bridge molecular discoveries and clinical implementation.

This Special Issue was conceived to address these challenges by bringing together contributions spanning molecular mechanisms, innovative diagnostic approaches, rare genetic diseases, congenital abnormalities, pediatric malignancies, and emerging therapeutic strategies. Rather than focusing exclusively on clinical observations, this Special Issue emphasizes studies incorporating molecular investigations capable of advancing mechanistic understanding and supporting future precision medicine approaches.

A prominent theme emerging from this Special Issue is the increasing importance of molecular characterization for improving diagnostic accuracy. Several contributions demonstrate how integrating genomic analyses with conventional clinical assessment facilitates earlier diagnosis and more accurate disease classification in case of congenital or acquired abnormalities. Another important aspect highlighted throughout this Special Issue is the growing role of translational research in pediatric medicine. Modern investigations increasingly combine experimental molecular techniques with clinical observations to identify disease mechanisms that may ultimately serve as therapeutic targets. Pediatric oncology is also a recurring topic, where advances in molecular biology continue to redefine disease classification and therapeutic decision-making in case of hepatoblastoma, neuroblastoma, or rhabdomyosarcoma. The studies included in this collection underscore the value of molecular profiling for identifying prognostic markers and potential therapeutic targets while emphasizing the necessity of integrating laboratory discoveries into multidisciplinary clinical care.

Several contributions additionally explore innovative diagnostic methodologies. Advances in next-generation sequencing, molecular imaging, laboratory biomarkers, and computational analyses have substantially improved clinicians’ ability to detect disease at earlier stages while reducing diagnostic uncertainty. Contemporary research increasingly combines detailed phenotypic characterization with comprehensive molecular profiling, recognizing that meaningful precision medicine requires integration rather than replacement of clinical expertise. This multidimensional approach represents one of the defining characteristics of modern pediatric research and is consistently reflected throughout the contributions assembled in this collection.

## 2. Addressing Current Knowledge Gaps

Although remarkable progress has been achieved in pediatric molecular medicine, substantial challenges remain before many of these advances can be routinely translated into clinical practice. The contributions collected in this Special Issue collectively illustrate both the achievements of contemporary research and the areas where further investigation is urgently needed. Future studies should integrate genomic sequencing with transcriptomic, proteomic, metabolomic, and functional analyses to improve diagnostic accuracy and better understand disease mechanisms. 

Several studies included in this Special Issue contribute to refining genotype–phenotype associations, but larger multicenter investigations involving diverse patient populations will be necessary to establish robust predictive models that support personalized clinical management. Continued efforts combining molecular biology with advanced computational analyses are expected to facilitate biomarker discovery and validation across multiple pediatric specialties. Functional studies using cellular models, patient-derived organoids, induced pluripotent stem cells, and animal models will remain essential for validating candidate therapeutic targets and understanding disease biology before clinical translation [5,6].

Future pediatric research must continue to address broader societal and ethical considerations associated with genomic medicine. Expanded genetic screening, incidental findings, data sharing, informed consent, and equitable access to advanced molecular diagnostics all represent important issues requiring ongoing attention as precision medicine becomes increasingly integrated into routine pediatric healthcare [7,8].

## 3. Conclusions

The articles collected in this Special Issue provide an excellent overview of the remarkable progress being achieved across pediatric molecular medicine. Together, they demonstrate how advances in genetics, molecular biology, developmental biology, and translational research are reshaping our understanding of congenital abnormalities, inherited disorders, pediatric malignancies, and other complex childhood diseases. Importantly, the contributions extend beyond describing clinical phenotypes by incorporating molecular investigations that enhance mechanistic understanding and support the development of innovative diagnostic and therapeutic strategies. Collectively, these studies emphasize that improving outcomes for children with complex diseases requires multidisciplinary collaboration, integration of molecular technologies with clinical expertise, and continued investment in translational research. While considerable challenges remain—including incomplete molecular diagnoses, limited functional validation of genetic variants, and the need for effective targeted therapies—the rapid pace of scientific innovation provides strong reasons for optimism. 

We hope that this Special Issue will stimulate further interdisciplinary collaborations among pediatricians, geneticists, molecular biologists, surgeons, oncologists, laboratory scientists, and bioinformaticians, ultimately accelerating the translation of molecular discoveries into meaningful improvements in pediatric patient care. Continued advances in precision medicine, supported by emerging technologies and international collaborative research, promise to transform the diagnosis and treatment of pediatric genetic diseases and congenital abnormalities, bringing increasingly individualized and effective healthcare to children worldwide.
